# The Medical Bulletin Visiting List

**Published:** 1891-01

**Authors:** 


THE MEDICAL BULLETIN VISITING LIST OR PHYSICIAN'S CALL RECORD. F. A. Davis, Publisher, Philadelphia (1231 Filbert Street) and London. 1891.

This visiting list is arranged upon a novel plan. It is at once a weekly and a monthly visiting list. The columns for the marks of visits made are arranged in groups of seven, representing, of course, a week. The four weeks are similarly represented, with an allowance for the extra days above four weeks that make a month. These weekly columns are placed upon pages one-half the width of the ordinary pages of the book; consequently, but one writing of the list of names is necessary for a whole month, as none of these described weekly accounts in any way cover up the list of names written at the beginning of the month. Of course, the book has a somewhat peculiar appearance when closed, because its thickness at the stitching is twice or three times what it is at the edges. But then it is exceedingly compact and fits the pocket well. There are pages for the special memoranda, such as births, deaths, etc. The prices are: 70 patients, daily, each month, $1.25; 105 patients, daily, each month, $1.50.

